# Colon cancer checks in when bile acids check out: the bile acid–nuclear receptor axis in colon cancer

**DOI:** 10.1042/EBC20210038

**Published:** 2021-11-26

**Authors:** Qin Tang, Ronald M. Evans

**Affiliations:** Gene Expression Laboratory, Salk Institute for Biological Studies, La Jolla, CA 92037, U.S.A.

**Keywords:** bile acid, colon cancer, colorectal cancer, FXR

## Abstract

Bile acids (BAs) are a class of hepatically derived metabolite-hormones with prominent roles in nutrient absorption, metabolic and immune homeostasis in the intestine. BAs are ligands for multiple nuclear receptors (NRs), through which they confer transcriptional regulation on target genes that form an enterohepatic hormonal feedback loop to regulate BA synthesis and maintain lipid homeostasis. Endogenous BAs made by the host undergo significant biotransformation by the gut microbiota in the intestine, which diversifies the intestinal BA pool and facilitate host–microbiota cross-talk through BA-mediated signaling. BAs dysregulation contributes to development of metabolic diseases, pathological inflammation and colon cancer. This review provides a brief historic perspective of the study of NR-mediated BA signaling transduction, with a focus on recent advancements in understanding the active role the gut microbiome plays in reshaping intestinal BA landscape, and the implications of novel microbially derived BAs in modulating immune homeostasis and cancer development in the host. Targeting the BA–NR signaling axis for pharmacological intervention provides ample opportunities in the prevention and treatment of intestinal diseases.

## Introduction

Colorectal cancer (CRC) affects over 140 000 patients annually in the United States and is currently the second leading cause of cancer-related deaths [1]. The etiology of CRC is complex, but research in the past few decades has clearly indicated that genetic, dietary and lifestyle factors affect development and progression of CRC. The most significant genetic factor in hereditary risk for CRC is loss-of-function mutations in the adenomatous polyposis coli (APC) tumor suppressor gene [2]. Studies dating back to the 1970s have reported that risk for developing CRC was much higher among Japanese individuals who converted into a low-fiber and high-fat ‘Western’ diet after immigration to the United States [3,4]. Since then, the prevalence of unhealthy dietary habits has been linked to rising incidence of CRC worldwide [5]. A recent alarming trend is that CRC risk is rapidly increasing (1–3% annually) in adults younger than 50 years in the United States [1]. In light of these evidence, the U.S. Preventative Services Task Force has issued a recommendation to start routine screenings for CRC at the age of 45, to increase early diagnosis and treatment, and curb CRC-related deaths [6].

In humans and mice, bile acids (BAs) are a class of steroid acids abundant in the bile. They are produced from cholesterol metabolism in the mammalian liver [7]. From 1950s to 1970s, research on the structure and function of BAs led by Hofmann [8] and others noted the amphipathic nature of BA molecules, and argued that they play a decisive role in facilitating lipid and vitamin absorption in the intestine [9]. In fact, BAs emulsify lipids so well that they have been medically administered in patients for dissolution of cholesterol stone in the gallbladder [10,11]. The 1980s saw an uptick in interest in BAs in the medical community, with reports implicating perturbations in BA metabolism in liver [12,13] and intestinal diseases [14,15] as well as cancer [16,17]. In the 1990s, major physiological functions of BAs were established. Briefly, BAs are synthesized in the liver, stored in the gallbladder and released into the small intestine through the bile duct. Following intestinal transit, most BAs are reabsorbed in the terminal ileum to the hepatic portal vein and trafficked back to the liver. Hepatic production of BAs and the total amount of BAs in enterohepatic circulation fluctuate significantly with respect to dietary lipid intake. BAs undergo enterohepatic circulation several times each day. Through enterohepatic circulation, BAs confer negative feedback regulation on lipid metabolism (in particular, cholesterol biosynthesis), and have pivotal roles in the regulation of glucose metabolism [18–22].

## A signaling molecule disguised as a detergent

In 1995, the Evans lab reported the isolation of an orphan receptor that forms a heterodimer with the retinoid X receptor (RXR) [23]. This work identified farnesol and related metabolites as potent activators of the complex. Farnesol is the chemical precursor to BAs, and the discovery in 1999 that BAs were potent farnesoid X receptor (FXR) ligands [24] redefined them as an unrecognized class of hormonal ligands. In the next few decades, research led by Chiang, Makishima et al., Russell and Setchell, Schoonjans et al. among others laid the foundation of contemporary understanding of BA physiology. There are a number of excellent reviews on this topic [25–30] that we encourage readers to seek out in order to get a panoramic view of research in this field. Very briefly, the first and only rate-limiting step in classic hepatic BA synthesis is catalyzed by the cholesterol 7α-hydroxylase (CYP7A1) that converts the cholesterol into 7α-hydroxycholesterol [31,32]. The liver also frequently conjugates BAs with glycine or taurine through a reaction catalyzed by the BA-CoA:amino acid N-acyltransferase (BAAT) prior to their release into the small intestine [33–35]. In humans, cholic acid (CA), chenodeoxycholic acid (CDCA) and their tauro- or glyco-derivatives make up the majority of BAs in circulation. There are two major classes of receptors for BAs in the intestine: the G-protein-coupled receptors (most notably TGR5 encoded by the *GPBAR1* gene) [36,37], or nuclear receptors (NRs) [24,38–40]. TGR5–BA interactions in health and disease have been extensively reviewed elsewhere [41,42]. In this review, we focus on NRs, a unique class of ligand-responsive transcriptional factors, which differs from surface receptors in their ability to enter the nucleus of the cell and mediate transcriptional regulation. Specifically, ligand-unbound NRs are typically retained within the cytosol; binding to ligands induces conformational changes in NRs and induce their translocation to the cell nucleus, where it binds at specific regulatory elements in the genomic DNA, and coordinate changes in transcriptional output of target genes through recruitment of transcriptional co-activators or co-repressors. Of multiple BA-responsive NRs reported in the literature, the FXR (encoded by NR1H4) is considered a master regulator of BAs in the gastrointestinal system.

**Figure 1:**
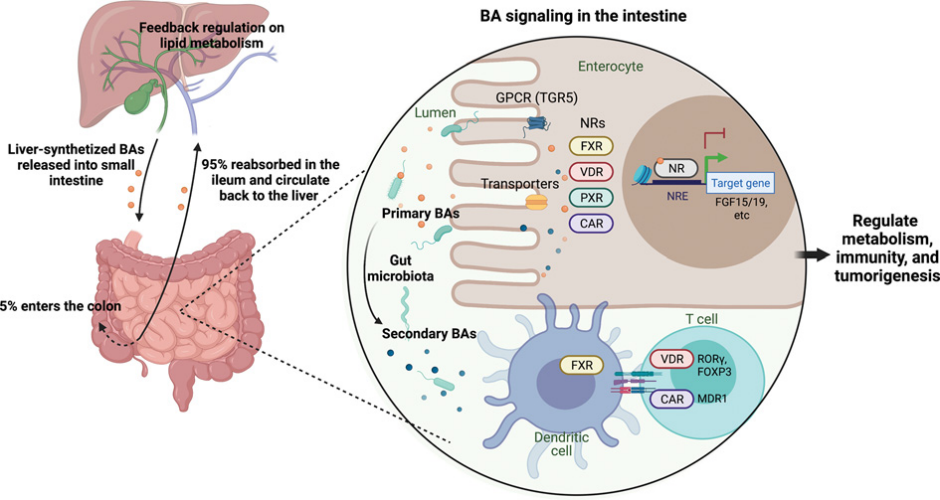
The enterohepatic circulation of BAs and signaling transduction through BA-responsive receptors in the intestine

**Figure 2:**
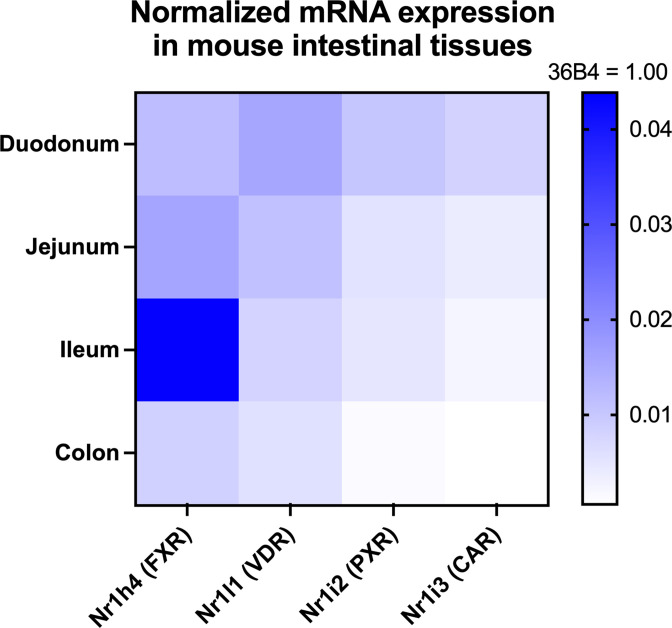
RNA expression of major BA-sensing NRs in the mouse intestine

Work in the late 1990s into the 2000s led by our group, Forman, Gonzalez, Mangelsdorf, Kliewer, Moore and many others uncovered that FXR is the cognate NR for CDCA [43], and revealed the molecular underpinnings of the BA–FXR signaling axis [44–49]. Briefly, cellular uptake of CDCA through the apical sodium bile salt transporter (ASBT) activates FXR in ileal epithelial cells, leading to its heterodimerization with RXR, translocation to the nucleus and binding to FXR response element (FXRE) therein. Transcription of two small peptide hormones, the human fibroblast growth factor (FGF) 19 (or its murine ortholog Fgf15), are up-regulated. FGF15/19 subsequently traffic back to the liver, initiating a signaling cascade involving the induction of small heterodimer partner 1 (SHP-1; NR0B2) and recruitment of liver receptor homolog 1 (LRH-1; NR5A2) to the CYP7A1 promoter [50,51]. This confers transcriptional repression of CYP7A1 and, consequently, mediates suppression of hepatic BA synthesis. Thus, BA levels are under negative-feedback control through the enterohepatic FXR-FGF15/19 signaling axis (Figure 1). In addition to FXR, other NRs including vitamin D receptor (VDR) [39], pregnane X receptor (PXR, encoded by NR1I2) [38], constitutive androstane receptor (CAR, encoded by NR1I3) [40] and the retinoic acid-related orphan receptor γ (RORγ, encoded by NR1F3) [52,53] can also respond to BAs, the details of which will be discussed later. Of these, NRs are differentially expressed in different segments of the gastrointestinal tract. Notably, FXR expression is the highest in the mouse ileum, consistent with the highest activity of BA reabsorption in this segment of the small intestine (Figure 2). Lastly, the liver X receptor (LXR), the master regulator of cholesterol metabolism in the liver, confers opposing effects of transcriptional regulation on CYP7A1 with FXR, constituting a dual network that balances the ‘yin’ and ‘yang’ of lipid metabolism [47,48]. The contemporary view holds BAs not only as molecular detergents that facilitate lipid absorption, but also metabolite-hormones that serve as signaling molecules in the digestive system [27,29].

## BA diversity, a gut microbiome production

In recent years, it has been increasingly appreciated that the gut microbiome is an integral part of intestinal homeostasis and health [54,55]. While the majority of hepatically derived BAs are reabsorbed and recycled in the ileum, a small proportion (5%) comes into contact with the gut microbiome in the distal ileum, cecum and colon. The microbiome confers substantial biotransformation on host liver-derived BAs; the resultant microbially derived BAs are termed ‘secondary’ BAs to differentiate from host-derived ‘primary’ BAs [56–58]. Human gut microbiota is dominated by *Firmicutes*, *Bacteroidetes* and *Actinobacteria* [59]. Many of these species have specialized enzymes to modify the cholesterol backbone or deamidate glycine or taurine-conjugated primary BAs [60,61]. One particular microbially catalyzed BA transformation is 7α-dehydroxylation, which produces dominant secondary BAs deoxycholic acid (DCA, from CA) and lithocholic acid (LCA, from CDCA) [62,63]. Recently, six new microbial enzymes necessary and sufficient for the conversion of CA into DCA have been identified [64], establishing a complete roadmap from a primary BA to its secondary BA derivative.

The gut microbiota changes swiftly in response to environmental factors [65], particularly to the influence of diet [66]. Dietary perturbation could alter the composition of the gut microbiome and disrupt the balance between beneficial and detrimental gut microbiota [67,68], resulting in impaired host metabolic and immune homeostasis [69] that can set the stage for disease development [70]. BAs can alter the gut microbiota composition by forming an intestinal microenvironment favorable to the survival and proliferation of certain microbes, at the expense of others. For example, obeticholic acid, a synthetic BA analog and FXR agonist, confers growth advantage on *Firmicutes* in the small intestine [71]. In inflammatory bowel disease (IBD) patients, the structure of the gut microbiota is significantly altered compared with healthy individuals [72,73], concurrent with reported disruption in BA metabolism [74]; both are thought to significantly contribute to pathogenesis and disease progression. Moreover, microbial biotransformation of BAs also significantly enriches the endogenous BA repertoire in the host [75]. Recent advancement in new techniques, particularly high-throughput LC–MS/MS mass spectrometry and 16S rRNA gene sequencing, allowed investigators to efficiently probe the diversity of secondary BAs in the mammalian gut. Capitalizing on these techniques, Dorrestein et al. [76] conducted comprehensive profiling of metabolome in the mammalian gastrointestinal tract. By comparing BA profiles in germ-free versus specific pathogen-free mice, they uncovered several previously unknown secondary BAs, including a unique class of phenylalanine or tyrosine-conjugated BAs on a cholate backbone [76]. Intriguingly, these novel secondary BAs activate FXR signaling *in vitro*, warranting further investigation on their functions in modulating the host BA–FXR signaling axis. The consensus now is that the gut microbiome actively participates in shaping the BA landscape in the mammalian gut, and regulate host health through the production of secondary BAs that engage host NRs to facilitate host–microbiome cross-talk.

Our deepening understanding of the gut microbiome and continuing characterization of secondary BAs also expanded our knowledge of BA-responsive NRs beyond FXR. Work by our group and the Kliewer group showed that VDR, PXR and its human homolog steroid and xenobiotic receptor (SXR) are receptors for LCA, one of the most abundant secondary BAs; activation of these NRs induces production of CYP3A in the liver to catabolize excess LCA, thereby conferring protection against liver toxicity [38,39,77]. VDR and PXR also participate in the enterohepatic BA–FGF15/19–CYP7A1 negative feedback loop to control BA synthesis in the liver [78,79] (Figure 1).

## At the crossroads of good and evil

The FXR gene has characteristics of a tumor suppressor in colon cancer. FXR knockout mice under carcinogen challenge had increased intestinal epithelial cell proliferation and tumor development [80]. In human CRC patients, low expression of FXR is correlated with poor prognosis [81]. By comparing human CRC samples, precancerous polyps and normal tissue, Bailey et al*.* showed that the NR1H4/FXR promoter is methylated in ∼12% of human colon cancer samples, contributing to epigenetic silencing of FXR expression in the early stages of human colon cancer development [82]. Together, these data supported that FXR down-regulation is an important event in CRC initiation, and suggested that activation of FXR in intestinal tissues could prevent or curb progression of colon cancer.

**Figure 3:**
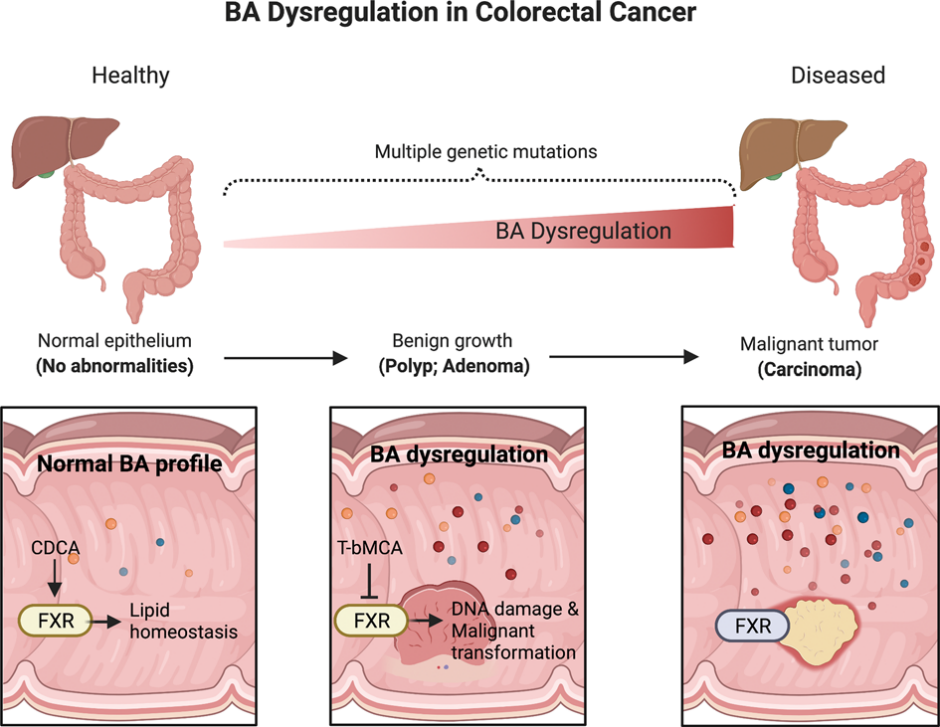
BA dysregulation and its implications in CRC development

BA dysregulation is a hallmark of CRC development [83]. Conventional wisdom dictates that BAs as surfactants are disruptive of the lipid bilayer of the cell membrane; therefore, aberrantly high levels of BAs are considered cytotoxic to intestinal epithelial cells and enterocytes. This notion is supported by multiple reports that prolonged exposure to aberrantly high levels of BAs poses elevated risk for the development of CRC [84–86]. Fecal levels of secondary BAs in human patients also correlate with mucosal and metabolic markers of CRC [87]. A recent study conducted by our group showed that, in CRC mouse models, HFD increases intestinal BAs as well as CRC incidence. Specifically, in the CRC (Apc^Min/+^) mouse model, HFD triggers a 60-fold increase in tauro-β-muricholic acid (T-βMCA, a secondary BA). T-βMCA was shown to be a potent antagonist of FXR in ileal epithelial cells as well as promoting DNA damage in Lgr5-expressing CRC stem cells. As a consequence of these changes, T-βMCA (like HFD) was sufficient to promote adenoma-to-adenocarcinoma transformation and accelerate CRC disease progression. Conversely, activation of FXR signaling by oral delivery of a gut-restricted FXR agonist (termed FexD), effectively curtailed tumor progression and prolonged survival [88]. This work lends support for the notion that the gut microbiome substantially modulates cancer development by the BA–NR signaling axis in the host, highlighting the potential of sustained FXR activation to curtail CRC development (Figure 3).

CRC develops at a unique site of peripheral immunity facilitated by localized chronic inflammation [89]. The gut-associated lymphoid tissue (GALT), including lamina propria (LP), intraepithelial lymphocytes, Peyer’s patches and the mesenteric lymph node, provides a prime interface with the gut microbiome as well as mediating immune education and tolerance [90,91]. Both FXR and TGR5 are expressed in intestinal and liver innate immune cell types [92]. Public data from the Immunological Genome Project [93] also confirmed expression of multiple NRs in T cells and innate lymphoid cells (ILCs) isolated from the mouse intestine. This provides a unique way for BA sensors in immune cells to impact intestinal immune homeostasis as well as intestinal carcinogenesis [94]. A number of secondary BAs that suppress FXR and PXR signaling in IBD patients during disease progression have been identified [95,96]. Habtezion et al. recently reported that *Ruminococcaceae*, one of few taxa known to include secondary BA-producing bacteria, was substantially depleted in ulcerative colitis (UC) patients [97]. Studies in mouse models showed that DCA, a secondary BA increased by Western diet, contributed to impairment of the intestinal barrier and exacerbated tumor progression in Apc^Min/+^ mice through FXR-dependent mechanisms [98,99].

Recently, a quartet of high-profile papers revealed diverse molecular mechanisms by which the microbial BA–NR axis confers regulation on intestinal immune homeostasis in the host. Song et al*.* showed that the abundant secondary BA LCA and its derivative 3-oxoLCA were required for induction of colonic RORγ+ Treg cells in mice. Taking a mouse genetics approach, they confirmed that VDR was required in this process, implicating the BA–VDR axis in the establishment of colonic immune tolerance [53]. Hung et al*.* also reported that two distinct derivatives of LCA, 3-oxo-LCA and isoalloLCA, affected intestinal T-cell lineage specification, albeit through different mechanisms. *In vitro* studies showed that 3-oxoLCA inhibited IL-17a^+^ Th17 cell differentiation by suppression of the lineage defining transcription factor RORγt, while isoalloLCA induced Foxp3^+^ Treg cell differentiation through mitochondrial redox-related mechanisms [52]. Campbell et al*.* reported that another secondary BA, 3β-hydroxydeoxycholic acid (isoDCA), increased Foxp3^+^ Treg cell induction in the colonic LP. Interestingly, this effect required the presence of antigen-presenting cells, i.e. dendritic cells (DCs) *in vitro*. Subsequent studies in DC-specific FXR knockout mice (Csf1r^Cre^Nr1h4^fl/fl^) confirmed that FXR signaling in DCs was indispensable for isoDCA-mediated Treg cell induction *in vivo*. The present study culminated in the design of an isoDCA-producing artificial microbiota capable of increasing the number of colonic Treg cells in mice [100]. Chen et al*.* showed that the nuclear xenobiotic receptor CAR (Nr1i3) also has a role in establishing mucosal immune cell tolerance of high BA concentrations in the ileum, the prominent site for BA reabsorption. *In vivo* studies showed that CAR coordinated up-regulation of the xenobiotic transporter MDR1 gene in CD4^+^ T effector cells in small intestinal LP to prevent BA toxicity and suppress inflammation [101]. Lastly, our group reported that pharmacological FXR activation suppressed production of pro-inflammatory cytokines IL17 and IL6 in type 3 ILCs (ILC3) in the small intestinal LP, and protected against intestinal inflammation in a chemically induced IBD mouse model [102]. In sum, these works highlight the roles of the BA–NR axis as a pivotal regulator for intestinal immune homeostasis.

In this review, we covered previously unexpected aspects of BA metabolism, their emerging roles in physiology, and offered an NR-centric view of the molecular mechanisms underlying response to BA-mediated signaling in the enterohepatic system. Multiple NRs, including but not limited to FXR, VDR, PXR, CAR and RORγ are concurrent sensors for BAs and critical components in an expansive signaling network that govern intestinal metabolic and immune homeostasis. Importantly, as NRs, they provide druggable targets for intervention in metabolic disorders and intestinal tumorigenesis. Understanding the dynamic interplay between dietary risk factors and the gut microbiota in healthy and diseased states hold promise for the development of novel methods for the prevention and treatment of CRC. Future work on secondary BAs and NR signaling could provide insight in biological mechanisms underlying host–commensal cross-talk and facilitate deeper understanding of how gut microbiome regulate host metabolism and immunity. Knowledge gained in these efforts could also guide the development of novel non-invasive approaches for engineering the commensal ecosystem to treat and prevent metabolic diseases and gastrointestinal cancers.

## Summary

- BAs are metabolite-hormones in enterohepatic circulation with prominent roles in lipid absorption, metabolic and immune homeostasis.
- The intestine harbors a diverse pool of host liver-derived as well as gut microbiome-transformed BAs, which serve as an unrecognized vehicle for host–microbiome cross-talk.
- BAs acting on innate and adaptive cell functions play important roles in maintaining immune homeostasis in the gut. BA dysregulation contributes to the inflammatory pathogenesis and drives tumorigenesis.
- The ligand-responsive nature of NRs provides unique opportunities pharmacological intervention in the treatment of diseases.

## Abbreviations

APC: adenomatous polyposis coli
BA: bile acid
CA: cholic acid
CAR: constitutive androstane receptor
CDCA: chenodeoxycholic acid
CRC: colorectal cancer
CYP7A1: cholesterol 7α-hydroxylase
DC: dendritic cell
DCA: deoxycholic acid
FGF: fibroblast growth factor
FXR: farnesoid X receptor
HFD: high-fat diet
IBD: inflammatory bowel disease
ILC: innate lymphoid cell
isoDCA: 3β-hydroxydeoxycholic acid
LCA: lithocholic acid
LP: lamina propria
NR: nuclear receptor
PXR: pregnane X receptor
RORγ: retinoic acid-related orphan receptor γ
RXR: retinoid X receptor
T-βMCA: tauro-β-muricholic acid
Treg: regulatory T cells
VDR: vitamin D receptor
